# 

**Published:** 1901-03-23

**Authors:** 


### NOTICE TO CORRESPONDENTS.

All MSS., Letters, Books for Review, and other matters intended for the Editor should be addressed The Editor, "The Hospital," The Hospital Building, 28 & 29 Southampton Street, Strand, W.C.

The Editor cannot undertake to return rejected MS., even when accompanied by stamped directed envelope.

**Notice to Advertisers and Subscribers.**

All Advertisements, Orders for Copies of The Hospital, and Business Communications should be addressed to **THE MANAGER (Not to the Editor),** The Hospital, 28 & 29 Southampton Street, Strand, London, W.C.

The Cost of Subscription, post free, is as follows:—Per week, 2½d.; per quarter, 2s. 8d.; per half-year, 5s. 3d.; per annum, 10s. 6d. Foreign:—Per annum, 15s. 2d., payable, in all cases, in advance.

**NOTICE.—Vol. XXVIII. of "The Hospital" (April to September, 1900), in handsome cloth binding, is now on sale at the Office of this Journal, price, 7s. 6d. Cases for binding the Numbers contained in this Volume 1s. 6d. Index to Vol. XXVIII., 2½d., post free.**

**The Monthly Part for April will be ready on April 1st. Price 6d., by post 8d.**

### BOOKS RECEIVED.

JOHN WRIGHT AND CO.

"'First Aid' to the Injured and Sick: An Ambulance Handbook." By F. J. Warwick, B.A., M.B., Cantab. M.R.C.S., L.S.A., and N. C. Tunstall, M.D., F.R.C.S.Ed.

"Sidmouth as a Health Resort and Place of Residence." By A. Macindoe, M.D., D.Ph.

"The Medical Annual, 1901." Nineteenth year.

LAMBERT PHARMACAL COMPANY.

"Medical and Surgical Experiences in the South African War."

NURSING NOTES OFFICE.

"Nursing Notes for 1900."

J. AND A. CHURCHILL.

"Diet and Food." By Alexander Haig, M.A., M.D.Oxon., F.R.C.P Third edition.

### Scraps and Gleanings.

The Earl of Rosebery has consented to become a vice-president of the Italian Hospital.

During 1900, 181 patients were admitted to the Luton Bute Hospital of whom 41 were free. During 1899 216 were admitted, of whom 88 were free.

The debt on the Cardiff Infirmary has now been wiped off. In a little over three months £11,000 has been collected, and as the debt was £10,700 the Infirmary is left with a sum of about £500 to the good.

The new wing at the Alexandra Hospital, Woodhall Spa, will shortly be completed, enabling the hospital to accommodate 30 instead of 20 patients as hitherto. These additions and improvements have cost £2,000 including furnishing.

The Hovis Bread Flour Company have issued a little booklet entitled "Pictorial London," price 1d., containing illustrations and short descriptions of well-known London places. It can be obtained of all newsagents, or from the Hovis Bread Company.

Out of 50,000 residents in Blackpool, there are fewer than one hundred individual annual subscribers to the hospital, apart from the members of the Board. The work of the hospital is, however, steadily growing, and in 1900 there was an increase in the number of patients of close upon 50 per cent. over 1899.

### The Student of Medicine.

**APPOINTMENTS.**

**E. S. Angove,** L.R.C.P.Edin., M.R.C.S.Eng., appointed District Medical Officer to the Flaxton Out-Relief Union. **S. T. Beckett,** L.R.C.P., L.R.C.S.Edin., appointed Certifying Factory Surgeon for the townships of Cullingworth, Harden, and Wilsden. **J. H. Bennett,** M.R.C.S., L.R.C.P.Lond., appointed Certifying Factory Surgeon for the Staplehurst District of Kent. **W. A. Bond,** M.A., M.D., D.P.H.Camb., appointed Medical Officer of Health for the Metropolitan Borough of Holborn. **C. J. A. Coates,** L.R.C.P., L.R.C.S. Edin., appointed Certifying Factory Surgeon for the Stokenchurch District of Bucks. **J. J. Cowan,** M.B.Edin., appointed District Medical Officer to the Upton-upon-Severn Union. **F. Graham Crookshank,** M.D.Lond., appointed Medical Officer of Health for the Urban District of Barnes, and Medical Superintendent of the Isolation Hospital, Barnes. **Alexander Day,** M.B., C.M.Aberd., appointed Certifying Factory Surgeon for the Wooler District of Northumberland. **E. L. D. Dewdney,** L.S.A., appointed District Medical Officer to the Williton Union. **G. H. Doudney,** M.B.Durh., appointed Certifying Factory Surgeon for the Wainfleet District of Lincolnshire. **Claude Douglas,** F.R.C.S.Eng., appointed Surgeon to the Leicester Infirmary. **T. Dunlop,** M.B., C.M.Edin., D.P.H.Camb., reappointed Medical Officer of Health to the Aldershot Urban District Council. **W. R. Edmond,** M.B.Edin., appointed Certifying Factory Surgeon for the Totnes District of Devon. **A. A. Edward,** M.D.Edin., appointed Certifying Factory Surgeon for the Borough of Rawtenstall. **C. N. Elliott,** M.B., appointed Medical Officer of Health to the Thrapston Urban District. **J. T. Farren,** L.R.C.S., L.R.C.P.Edin., appointed Medical Officer, and Medical Officer of Health for the Ardava District of the Glenties Union. **James Fletcher,** M.B., C.M. Aberd., appointed Resident Medical Officer to the Bristol City Fever Hospital. **George PocockGoldsmith,** M.D.Durh., M.R.C.S.Eng., appointed Consulting Physician to the Bedford County Hospital. **T. E. Gordon,** M.D.Durh., appointed Certifying Factory Surgeon for the Towyn District of Merioneth. **Francis J. Lander,** appointed Honorary Surgeon to the Nottingham General Dispensary. **T. C. Lawson,** M.R.C.S.Eng., appointed Certifying Factory Surgeon for the Sherston District of Wilts. **T. C. Lawson,** M.R.C.S.Eng., appointed Medical Officer for the Fourth District of the Malmesbury Union. **A. R. McLachlan,** A.R., M.R.C.S., L.R.C.P.Lond., appointed Assistant Medical Officer of the Infirmary and Schools of the Brentford Union. **H. A. Marquis,** L.R.C.P., L.R.C.S.Edin., appointed District Medical Officer to the North Bierley Union. **Cecil Marriott,** M.B., B.C.Camb., appointed Assistant Surgeon to the Leicester Infirmary, *vice* **C. Douglas,** F.R.C.S.Eng. **George Myles,** L.R.C.P., L.R.C.S.Edin., appointed Certifying Factory Surgeon for the Limerick District. **Wm. Palmer,** M.R.C.S., L.P.C.P. Lond., appointed Certifying Factory Surgeon for the Linton District of Cambridgeshire. **Charles Parker,** M.B., Ch.M. Edin., appointed Honorary Medical Officer to the Launceston General Hospital, Tasmania. **Edwin Peacock,** M.R.C.S. Eng., appointed Certifying Factory Surgeon for the Nuneaton District of Warwickshire. **A. Pearson,** appointed Medical Officer to the Clifton District of the Newark Union. **George B. Pemberton,** M.B., Ch.B.Edin., appointed House Surgeon to the District Infirmary, Ashton-under-Lyne. **F. B. Wilmer Phillips,** M.A., M.D.Oxon., B.Sc.Lond., M.R.C.S.Eng., appointed Physician to the Bedford County Hospital. **F. A. Pitts-Tucker,** M.R.C.S., L.R.C.P.Lond., appointed District Medical Officer to the Ely Union. **J. M. Roe,** M.B. Syd., appointed Surgeon to the Hospital at Mareeba, Queensland. **F. J. Sadler,** M.B., B.Ch. Oxon., appointed Certifying Factory Surgeon for the Barnsley District of the West Riding of York. **J. Scarr,** L.R.C.P., L.R.C.S.Edin., appointed Medical Officer to the Rochdale Union Cottage Homes. **J. C. Shand,** M.B.Glasg., appointed Government Medical Officer at Penrith, New South Wales. **A. J. Sharp,** M.D. Lond., appointed Surgeon and Agent to H.M. Coastguard and Royal Naval Reserve of the Whitby Division. **Edward Colby Sharpin,** L.R.C.P.Edin. and L.M., M.R.C.S.Eng., appointed Consulting Surgeon to the Bedford County Hospital. **H. L. A. Shorter,** M.B., M.Ch.Syd., appointed Medical Officer at Mount Garnet, Queensland. **H. P. Sleigh,** M.B., C.M.Aberd,, appointed House Physician to the Leeds General Infirmary. **H. Hammond Smith,** L.R.C.P.Lond., M.R.C.S.Eng., appointed Certifying Factory Surgeon for the Uxbridge District of Middlesex. **T. Telford Smith,** M.D., appointed Certifying Factory Surgeon for the Wimborne District of Dorset. **W. D. Stamp,** L.R.C.P.Edin., M.R.C.S. Eng., appointed Certifying Factory Surgeon for the Plympton District of Devonshire. **W. C. Steele,** M.B., C.M.Glasg., appointed Certifying Factory Surgeon for the Carshields District of Northumberland. **W. W. Stevens,** M.B., Ch.M.Syd., appointed Junior Medical Officer in the Lunacy Department, New South Wales. **C. J. Taylor,** M.B.Syd., appointed Medical Officer at Georgetown, Queensland. **Attwood Thorne,** M.B.Lond., appointed Assistant Surgeon to the London Throat Hospital, Great Portland Street, W. **Hubert Tibbitts,** M.B.Edin., M.R.C.S.Eng., appointed Medical Officer to H.M. Prison, Warwick. **W. E. F. Tinley,** M.D.Durh., appointed Medical Officer of Health to the Whitby Urban District Council. **Fredk. T. Travers,** M.B., B.S.Lond., F.R.C.S.E., appointed Surgeon to the West Kent General Hospital, Maidstone. **L. J. Willan,** M.R.C.S.Eng., appointed Certifying Factory Surgeon for the Dorking District of Surrey. **T. Kenway Williams,** M.R.C.S., L.R.C.P.Lond., appointed Medical Officer to the Dudley Dispensary. **L. E. Wood,** M.B.Lond., M.R.C.S.Eng., appointed Certifying Factory Surgeon for the Penshurst District of Kent

